# The Effects of Resistance Exercise With Blood Flow Restriction on Flow-Mediated Dilation and Arterial Stiffness in Elderly People With Low Gait Speed: Protocol for a Randomized Controlled Trial

**DOI:** 10.2196/14691

**Published:** 2019-11-01

**Authors:** Samuel Amorim, Hans Degens, Alexandra Passos Gaspar, Luciana Diniz Nagem Janot De Matos

**Affiliations:** 1 Hospital Israelita Albert Einstein São Paulo Brazil; 2 Research Centre for Musculoskeletal Science & Sports Medicine Manchester Metropolitan University Manchester United Kingdom; 3 Lithuanian Sports University Kaunas Lithuania; 4 University of Medicine, Pharmacy, Science and Technology of Targu Mures Targu Mures Romania

**Keywords:** blood flow restriction, arterial stiffness, elderly people

## Abstract

**Background:**

During aging, a significant loss of muscle mass, strength, and power is associated with a decline in daily functional capacities. Traditionally, resistance training is prescribed to prevent or reverse the skeletal muscle weakness, but the required training intensity may be too demanding for older people with poor physical performance. Resistance exercise with blood flow moderation (KAATSU training), originally developed in Japan, combines resistance exercise with blood flow restriction. It has been reported that KAATSU training enhances muscle hypertrophy in many populations. However, few studies have evaluated the effects of resistance exercises with blood flow restriction in elderly people and how this affects vascular structure and function.

**Objective:**

The aim of this study was to evaluate (1) the acute and chronic effects of resistance exercise with blood flow restriction on vascular health in elderly people with low gait speed and (2) whether low-load resistance training with blood flow restriction elicits similar strength and gait speed gains to those elicited by conventional resistance training without blood flow restriction.

**Methods:**

This is an ongoing randomized controlled trial in elderly people with low gait speed. Overall, two study arms of 13 participants each perform resistance exercise with and without blood flow restriction. The 2 groups are as follows: the control group will perform conventional resistance exercise (60% of 1 repetition maximum) and the KAATSU group will perform the low-load resistance exercise with blood flow restriction (20% of 1 repetition maximum) for 12 weeks. Pulse wave velocity, venous occlusion plethysmography, and flow-mediated dilation are used to assess arterial stiffness, muscle blood flow, and endothelial function, respectively. The secondary outcomes are gait speed, strength, and quality of life. All measures will be performed before and after the training program.

**Results:**

This research study is in progress. Recruitment has started, and data collection is expected to finish in August 2020.

**Conclusions:**

The findings of this study will have important implications for the rehabilitation of elderly people.

**Trial Registration:**

ClinicalTrials.gov NCT03272737; https://clinicaltrials.gov/ct2/show/NCT03272737

**International Registered Report Identifier (IRRID):**

DERR1-10.2196/14691

## Introduction

### Background

According to the World Health Organization, many countries, including China, Thailand, and Brazil, will see an increase in the percentage of the population over 65 years, from 7% in 2000 to 14% in the 2030s [1].

This increase in the proportion of older people represents a huge challenge for health care as aging is accompanied by decrements in cardiovascular function, skeletal muscle weakness, and changes in blood coagulation, all of which have a negative impact on the functional capacity of the elderly person [2,3].

Therefore, strategies that can prevent, minimize, or even reverse these effects of aging are imperative to maintain mobility and the ability to perform activities of daily living that are fundamental for the autonomy of elderly people [4].

### Senescence and Skeletal Muscle System

One of the major factors contributing to the age-related muscle wasting is disuse; however, even master athletes show aging-related muscle wasting and weakness, suggesting that other factors, such as an inherent aging process, must also contribute [5].

Elderly people with muscle weakness have 2.6 times higher risk of severe mobility limitation, 4.6 times higher risk of low gait speed, and 2.1 times higher risk of mortality compared with elderly people without muscle weakness [6].

### Senescence and the Cardiovascular System

Progressive loss of cardiomyocytes, reduction of the β_1_-adrenergic response, and degenerative alterations in the sinus node are all hallmarks of the aging heart. In parallel, there is an increased deposition of collagen that will contribute to diastolic dysfunction of the heart [7].

In addition to these cardiac changes, aortic stiffness is increased and atheromatous plaques and endothelial dysfunction develop [8]. Levels of coagulation factors are elevated and those of anticoagulants are decreased, increasing the underlying incidence of thrombosis, especially above the age of 70 years [9,10].

### Physical Exercise and the Cardiovascular System

Many questions related to the effect of physical training on the health of older people still need to be answered, such as the most appropriate type and the best form of training to enhance functional gains without secondary vascular impairment. Aerobic training improves the cardiovascular system, increases muscle blood flow, arterial compliance, and endothelial function that reduce the risk of comorbidities [11]. However, the use of this exercise modality in elderly people with reduced gait speed is limited [12].

Resistance exercise is a potent tool to gain and maintain strength and skeletal muscle mass in sarcopenic older people with diminished gait speed [13,14], but high intensities (>60% of 1 repetition maximum [RM]) are required for gains in skeletal muscle mass [15]. However, older people may not be able to cope with such loads and should avoid this type of exercise as it may cause undue stress on the cardiovascular system [16].

Recently, it was reported that low-intensity resistance exercise with blood flow restriction (BFR) may be able to induce gains in strength and muscle mass in older people [17]. Typically, BFR training uses low loads (20%-30% of 1RM) and short periods of training [18], a load that is better coped with and may thus be more applicable to elderly people, especially those who are sarcopenic. However, so far there are no studies evaluating the safety of BFR training for the cardiovascular system in this population [19].

### Study Aim and Hypotheses

The aim of this study is to evaluate (1) the acute and chronic effects of resistance exercise with BFR on vascular health in elderly people with low gait speed and (2) whether low-load resistance training with BFR elicits similar gait speed and strength gains to those elicited by conventional resistance training without BFR. Specifically, we will investigate the effect of the 2 training modalities on arterial stiffness, muscle blood flow, endothelial function, gait speed, and muscle strength in this population.

Our hypotheses are that low-load resistance exercise with BFR elicits the same increase in strength and improvement in quality of life as conventional resistance training and that it has no detrimental effect on arterial stiffness, muscle blood flow, and endothelial function. The findings of this trial may inform future recommendations for training of the elderly population.

## Methods

### Study Design

This study is designed as a randomized, blind, controlled intervention trial.

### Ethical Approval

The study received ethical approval from the ethical committee of the Hospital Israelita Albert Einstein and is registered on the ClinicalTrials.gov website. Participants are only included after signing the informed consent form.

Identifiable elements, including names, phone numbers, street addresses, city or state, zip code, email addresses, and date of birth will be collected but maintained under strict confidentiality. Screening materials will be kept for the participants recruited in the study and destroyed for those that do not meet the criteria or decide not to take part. Authorization for use and disclosure of the participants’ personal health information is restricted to this specific study, and data will be kept for 5 years after publication.

### Sample Size and Power Calculation

The sample size was calculated with Stata software (StataCorp LP) for both the acute and chronic protocols based on previous reports. It has been reported that flow-mediated dilation (FMD) was on average 4.3% (SD 3.1%) and arterial stiffness was on average 9.2 m/s (SD 1.1 m/s) [20].

Power analysis indicated that we need 10 participants per group to detect a difference in FMD of 3.1% and arterial stiffness on average 1.1 m/s between groups at a statistical power of 0.80 and an alpha of .05.

Owing to the conditions and frailty of elderly people, up to 20% dropouts are expected, and therefore, we are recruiting 13 participants per group.

### Eligibility

The eligibility criteria for participation in the study are provided in Textbox 1.

**Inclusion and exclusion criteria.**

Inclusion criteria:

- Elderly people aged ≥65 years
- Gait speed <0.9 m/s

Exclusion criteria:

- Smoking <6 months
- Previous deep venous thrombosis
- Uncontrolled arterial hypertension (blood pressure >160/100 mm Hg)
- Uncontrolled dyslipidemia (total cholesterol >220 mg/dL)
- Infections within the past 1 month
- Osteoarticular or neurological problems that prevent training
- Use of oral anticoagulants
- Symptomatic peripheral obstructive arterial disease or ankle-brachial index <0.9
- History of anemia, cerebrovascular disease, or myocardial infarction in the last 6 months
- Elderly people with uncontrolled diabetes mellitus or peripheral neuropathy
- Use of double antiplatelet agents: aspirin, Aggrenox, cilostazol, eptifibatide, ticlopidine, and tirofiban

### Participant Information and Informed Consent

Eligible participants are recruited primarily based on the results of a gait speed test. They consult with a cardiologist and a physiatrist to assess their health and determine whether they can participate in the study. In addition, medical records of the participants will be filled in for later analysis. Eligible participants are informed of their rights, and the procedures of the study and will be allowed to participate only if they provide written informed consent. Relatives are informed about the details of the tests and exercise sessions, if necessary.

### Recruitment

Participants are recruited from the Vila Mariana Unit of the Hospital Israelita Albert Einstein and elderly care centers in São Paulo.

### Randomization

Using a website [21], participants are randomized into two groups, each with 13 participants:

1. Low-load resistance exercise group (20% of 1RM) with BFR (resistance exercise with blood flow moderation, KAATSU);
2. Conventional resistance exercise (CRE) group (60% of 1RM).

The block sizes and randomized sequences are hidden from those who recruit or allocate participants to prevent predictability of the next assignment [22]. The participants must be aged 65 years or above.

### Blinding

Research team members administering clinical assessments are blinded from participant allocation. Participant allocation will not be revealed during the study.

### Exercise and Training Intervention

#### Acute Protocol: Resistance Exercise Session

Participants are instructed to consume a light meal before the training sessions and drink water. They should avoid physical activity and alcohol consumption 48 hours before the sessions. In addition, 24 hours before the first clinical tests, they should avoid consuming caffeine, chocolate, and tea.

Before the experimental exercise session, all participants undergo 2 familiarization sessions and 1RM tests to standardize the resistance exercise intensity. The KAATSU group participants perform bilateral seated knee extension and leg press exercises: 3 sets of 15 repetitions with 20 seconds rest between sets and 60 seconds between exercises at 20% of 1RM (Table 1). There is a 72-hour rest interval between sessions. Then, cuffs are inflated to a pressure that does not cause pain or discomfort during the warm-up and resistance exercises. This protocol is based on the study that evaluated KAATSU training in patients with metabolic diseases [23].

The CRE group individuals perform bilateral seated knee extension and leg press exercises: 3 sets of 15 repetitions with 60 seconds of rest between both sets and exercises, at 60% of 1RM.

**Figure 1:**
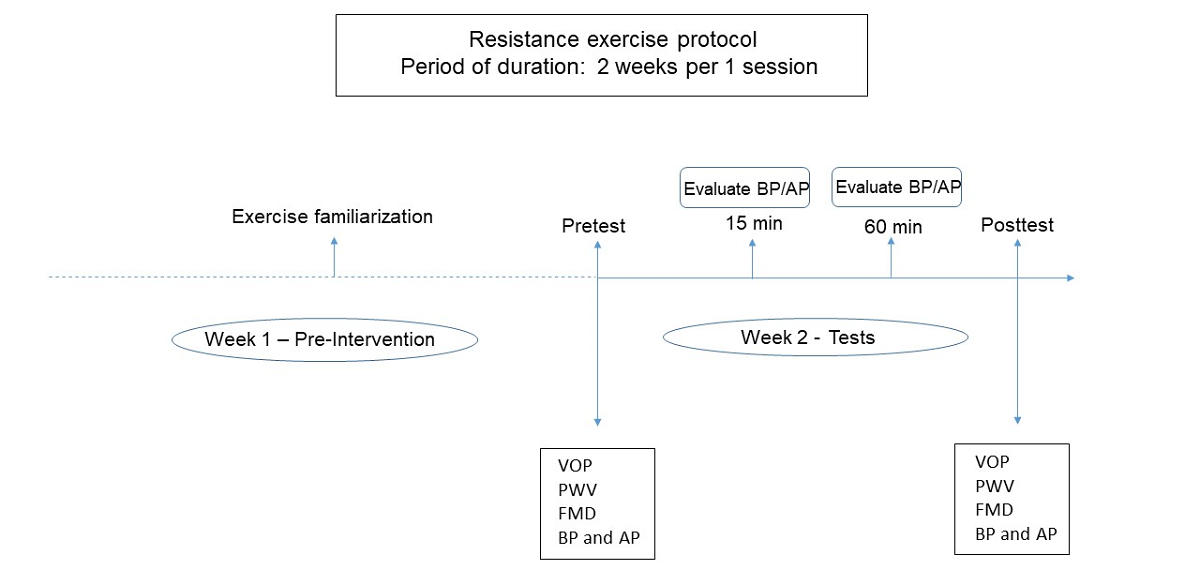
The sequence of activities that will be developed during the acute study. AP: arterial pressure; BP: blood pressure; FMD: flow-mediated dilation; PWV: pulse wave velocity; VOP: venous occlusion plethysmography.

Blood pressure and heart rate are evaluated before and 15 min, 30 min, and 60 min after the acute exercise session. Arterial stiffness, muscle blood flow, and vascular endothelial function are measured before and 60 min after the exercise session (Figure 1).

**Table 1:** Resistance exercise protocol of the acute study (session 1).

Groups	Series	Repetitions	Exercises	% of 1 repetition maximum	Lifting cadence (s)	Interval between sets (s)	Session duration (min)	Cuff size (mm)
Conventional resistance exercise	3	15	KE^a^ and LP^b^	60	1-1^c^	60	10	—^d^
KAATSU	3	15	KE and LP	20	1-1	20	10	50

^a^KE: knee extension.

^b^LP: leg press.

^c^1-1: 1.0 s concentric and 1.0 s eccentric lifting cadence.

^d^Not applicable.

#### Chronic Protocol: Resistance Training Program

**Figure 2:**
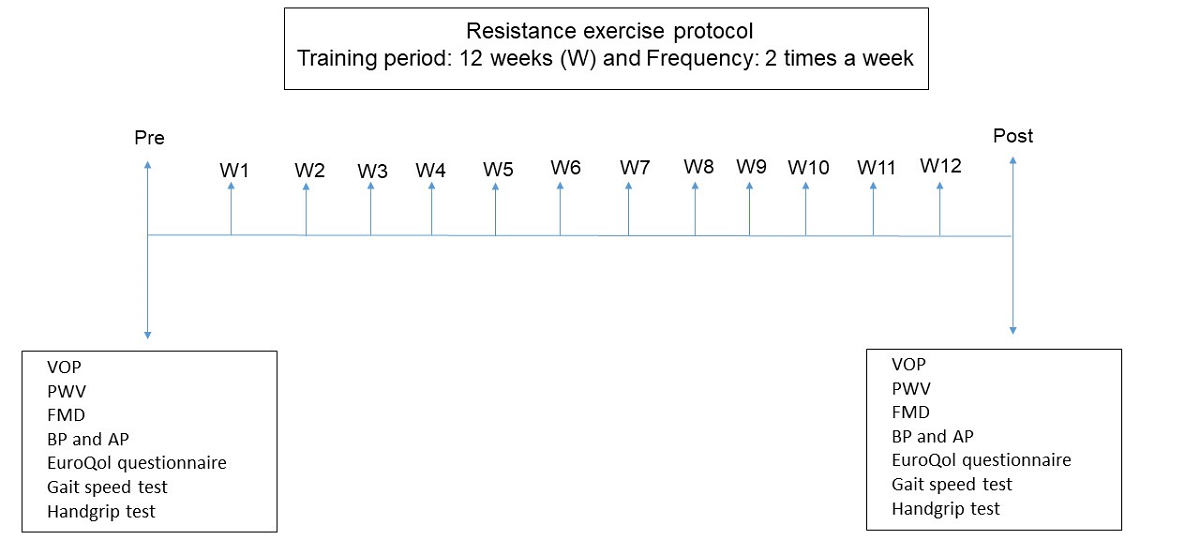
The sequence of activities that will be developed during the chronic study. AP: arterial pressure; BP: blood pressure; FMD: flow-mediated dilation; PWV: pulse wave velocity; VOP: venous occlusion plethysmography.

The protocol comprises 2 sessions per week for 12 weeks (Figure 2).

All groups are submitted to the same exercise protocol. The KAATSU group participants perform 2 sets of 15 repetitions at 20% of 1RM each time. The CRE group participants perform 2 sets with 15 repetitions at 60% of 1RM for each exercise in the first 4 weeks. The rest interval between exercises is 60 seconds for both groups, and rest interval between sets is 60 seconds for the CRE group and 20 seconds for the KAATSU group. The exercise duration of each repetition is 2.0 seconds (1.0 second concentric and 1.0 second eccentric lifting cadence). The exercise volume is increased to 3 sets for both groups in the fifth week of training. A load adjustment is carried out in training sessions 9 and 18 (Table 2).

**Table 2:** Resistance exercise protocol of the chronic study.

Sessions	Groups	Series	Repetitions	Exercises	% age of 1RM^a^	Lifting cadence (s)	Interval between sets (s)	Session duration (min)	Cuff size (mm)
1-8	CRE^b^	2	15	KE^c^ and LP^d^	60	1-1^e^	60	10	—^f^
1-8	KAATSU	2	15	KE and LP	20	1-1	20	10	50
9	CRE and KAATSU	—	—	—	1RM test	—	—	—	—
10-17	CRE	3	15	KE and LP	60	1-1	60	10	—
10-17	KAATSU	3	15	KE and LP	20	1-1	20	10	50
18	CRE and KAATSU	—	—	—	1RM test	—	—	—	—
19-24	CRE	3	15	KE and LP	60	1-1	60	10	—
19-24	KAATSU	3	15	KE and LP	20	1-1	20	10	50

^a^RM: repetition maximum.

^b^CRE: conventional resistance exercise.

^c^KE: knee extension.

^d^LP: leg press.

^e^1-1: 1.0 s concentric and 1.0 s eccentric lifting cadence.

^f^Not applicable.

### Determination of Blood Flow Restriction Pressure

BFR is accomplished using the KAATSU Nano device (KAATSU Global) that automatically detects the pressure needed on the limbs to reduce blood flow.

First, baseline pressure is calculated according to the age and general physical condition of the participants. This is the pressure observed after manually tightening the pneumatic cuffs on the upper arms or upper legs. Second, the cuffs are placed around both upper limbs, and a cycle function is started that comprises 8 cycles of 20 seconds inflation and 5 seconds deflation of the cuffs. After this step, the instructor removes the cuffs and places the leg cuffs on both lower limbs and inflates the cuffs up to the optimal pressure that does not cause pain or discomfort. The optimal pressure values result from a combination of age, level of fitness, limb circumference, and tests standardized by the methodology [24]. Participants remain with the cuffs on the lower limbs from the beginning to the end of the exercise session [23].

### Data Management

Data from the trial are routinely scrutinized for omissions and errors. All manually entered data are entered twice, and the source of any inconsistency is explored and resolved. Electronic data are stored and copied to an external hard drive. Data are only accessible to the study researchers. Each participant in the study is provided with an identification number, and the recorded data are coded using this number.

### Primary Goals

#### Venous Occlusion Plethysmography Protocol

Muscle blood flow is evaluated with venous occlusion plethysmography. The participant’s legs are elevated above the level of the heart to ensure adequate venous drainage. A mercury-filled silastic tube, connected to a low-pressure transducer and a plethysmograph (DE Hokanson), is placed around the largest circumference of the calf region. One cuff is placed around the ankle and another around the thigh. The ankle cuff is inflated to a suprasystolic pressure, 30 seconds before starting the measurements. At 15-second intervals, the cuff around the thigh is inflated above the venous pressure for a period of 7 to 8 seconds. Increased tension in the silastic tube reflects an increase in leg volume and vasodilation. The signal of the muscle blood flow wave is recorded on a polygraph and analyzed every minute, averaging 3 records per minute. The protocol is performed in a cycle of 5 min of rest, 3 min of isometric exercise, and 2 min of recovery [25].

#### Isometric Handgrip Exercise Protocol

In a supine position, the maximal voluntary handgrip force is determined as the highest force in 3 consecutive attempts using a Jamar hydraulic palmar dynamometer (Asimow Engineering). For the activation of the central command, mechanoreceptors, and muscular metaboreceptors, the individual performs, after 5 min of rest (baseline records), 3 min of exercise at 30% of maximal voluntary contraction. This maneuver isolates the activation of muscle metaboreceptors to observe their selective activation [26]. After the isometric handgrip exercise, there are 2 min for recovery to occur. Throughout the protocol, muscle blood flow, blood pressure, and heart rate are recorded.

#### Measurement of Arterial Stiffness—Pulse Wave Analysis and Velocity

Arterial stiffness is estimated from the carotid-femoral aortic pulse wave velocity [27]. The carotid-femoral aortic pulse waves are recorded by tonometry (SphygmoCor, AtCor Medical). At the same time, an electrocardiogram is registered to calculate the wave transit time. Overall, 2 distances are measured: the recording point between the carotid artery and the sternal furcula (distance 1), and between the sternal furcula and the recording point in the femoral artery (distance 2). The distance traveled by the pulse wave is calculated as *distance 2*−*distance 1*. The carotid-femoral aortic pulse wave velocity is calculated as follows: carotid-femoral aortic pulse wave velocity=¼(×)distance traveled by the pulse wave (m)/transit time (seconds).

#### Basal Blood Flow and Vasodilatory Capacity—Flow-Mediated Dilation

Basal blood flow and flow-mediated vasodilation are measured as previously described [28,29]. Images of the brachial artery are recorded by a 2-dimensional ultrasonography device with a spectral Doppler and linear transducer (Ultra-0122, Philips).

The participant is maintained in the supine position with the arm slightly abducted, for 20 min. After locating the brachial artery, the transducer is placed on the anteromedial aspect of the arm, perpendicular to the axis of the arm, 2 cm to 10 cm above the antecubital fold, over the artery. To confirm the location and quality of the arterial pulse obtained, Doppler is triggered. The resolution of contrast, depth, and gain is adjusted to optimize the longitudinal images of the lumen and arterial wall interface. The insonation angle is ≤60°. Blood flow velocity spectra are recorded simultaneously in the 10-MHz linear pulse mode. The diameter of the artery and the basal blood flow are recorded continuously over 120 seconds. After the baseline recording, a cuff on the forearm is inflated to 50 mm Hg above the systolic blood pressure. The occlusion is maintained for 5 min and then released quickly. The Doppler recordings are resumed 30 seconds before deflating the cuff and are continued for another 180 seconds. The diameter and the postocclusion blood flow are measured after release. The vasodilatory capacity is calculated as the percentage increase in the diameter of the brachial artery after occlusion.

### Secondary Goals

#### Speed Gait Test

To measure their gait speed, participants walk 4.6 m and the time needed to cover this distance is recorded. The mean of 3 attempts is calculated and divided by the distance. The participants included must achieve an average of <0.9 m/s in the walking test [30,31].

#### One Repetition Maximum Test (Knee Extension and Seated Leg Press)

The dynamic force of the lower limb muscles is evaluated by the maximal repetition of knee extension and leg press exercise, according to the protocol presented in a previous study [32]. The partipants perform a warm-up comprising 1 series with 10 unloaded repetitions. After this step, the mass to be lifted is progressively increased until the maximum load that can be lifted is reached, with a maximum limit of 5 attempts and a 3 min to 5 min interval between them. The test is conducted by a physical education professional who verbally encourages the participants throughout these steps and performs the load adjustment at weeks 5 and 10.

#### Heart Rate and Blood Pressure

Blood pressure and heart rate are evaluated with oscillometry (Dixtal DX 2020).

#### Anthropometric Assessment

Anthropometric measurements are registered before and after the training program following the International Society for Anthropometric Assessments standards [33]. Body mass is measured with an accuracy of 0.1 kg (Filizola). Stature is obtained by means of a stadiometer with an accuracy of 0.5 cm. Body mass index is calculated as body mass divided by height squared. The circumference of the quadriceps is measured using a tape measure (Seca) with a precision of 0.1 cm.

#### Quality of Life EuroQol-5 Domain

The questionnaire Quality of life Euro QoL–5 Domain s used to estimate the quality of life in participants before and after the study [34].

### Benefits

The possible benefits of KAATSU training are improved muscle strength and gait speed in elderly people, without any detrimental effect on arterial stiffness, muscle blood flow, or endothelial function, and perhaps even an enhanced vascular function, all achieved at a lower training load with BFR than with conventional resistance training.

### Adverse Events and Risks

BFR exercise may cause headache, red spots, redness, pain, and discomfort in lower limbs during or after exercise sessions.

### Auditing

Auditing is carried out as per the policies of the sponsor and of those overseeing the sponsor.

## Results

The last equipment for the study was obtained in January 2018. Recruitment and data collection were initiated in February 2018. Data collection is expected to be completed in January 2020, and the results are expected to be available in August 2020.

## Discussion

### Overview

This is the first time that the effects of BFR exercise on elderly people with low gait speed are evaluated utilizing the gold standard cardiovascular assessment. All necessary support and funding to conduct our study have been received.

Studies that have analyzed the effects of BFR with resistance exercise on cardiovascular function in older people are scarce. Shimizu et al showed that BFR training improved endothelial function and blood circulation in active elderly people [35]. Similarly, Patterson et al reported that 4 weeks of unilateral plantar flexion with BFR enhanced limb blood flow in sedentary older people [36]. Yasuda et al demonstrated that 3 months of low-intensity resistance exercise with BFR did not change arterial stiffness and muscle blood flow in inactive seniors [37]. So far, there is thus no indication that BFR exercise has detrimental effects on vascular function, but no studies have been performed on elderly people with poor physical performance. Therefore, studies are necessary to assess the benefits and possible harm for cardiovascular function owing to BFR exercise in older people.

This protocol describes a collaborative co-design study focused on the application of a new treatment method to combat sarcopenia in the older population. The findings from this study will have important implications on the safety to prescribe this type of exercise to the elderly people.

### Limitations

Finding eligible participants may be problematic, and the exclusion criteria limit our investigation in elderly people with low gait speed.

### Conclusions

This trial will evaluate the effects of resistance training with or without BFR on muscle strength and gait speed and whether BFR during exercise has any detrimental or beneficial effects on arterial stiffness, muscle blood flow, and endothelial function in elderly people with low gait speed.

## Abbreviations

BFR: blood flow restriction
CRE: conventional resistance exercise
FMD: flow-mediated dilation
RM: repetition maximum
